# Psychological distress and caregiver burden among informal caregivers of patients with earthquake-related and stroke-related dependency: a comparative study

**DOI:** 10.3389/fpsyt.2026.1871183

**Published:** 2026-06-22

**Authors:** Emine Çetin Duru, Barış Kılıç Demir, Alper Uysal

**Affiliations:** 1 Adana City Education & Research Hospital Physical Medicine & Rehabilitation Department, Adana, Türkiye; 2 Adana City Education & Research Hospital Psychiatry Department, Adana, Türkiye; 3 Mersin City Education & Research Hospital Physical Medicine & Rehabilitation Department, Mersin, Türkiye

**Keywords:** anxiety, caregiver burden, depression, earthquake, informal caregivers, post-traumatic stress disorder, social support, stroke

## Abstract

**Objective:**

This study aimed to compare the psychological burden of informal caregivers of patients with earthquake-related dependency and those caring for patients with stroke-related dependency following the 6 February 2023 Türkiye–Syria earthquakes.

**Methods:**

A total of 90 informal caregivers of dependent patients were included in this comparative study: 60 caregivers of stroke patients and 30 caregivers of patients with earthquake-related dependency. Participants were assessed using the Beck Depression Inventory, Beck Anxiety Inventory, Beck Hopelessness Scale, Caregiver Burden Scale, and the PTSD Checklist for DSM-5 (PCL-5).

**Results:**

Caregiver burden was significantly higher among male caregivers (p=0.026). Caregivers of patients with earthquake-related dependency more frequently reported receiving external support than caregivers of stroke patients (p=0.004). Overall, caregiving was perceived as a moderate burden in both groups. The distributions of depressive symptoms, anxiety symptoms, and hopelessness levels did not differ significantly between the groups (p=0.172, p=0.172, and p=0.834, respectively). Among caregivers of stroke patients, PTSD symptoms and caregiver burden showed moderate positive correlations with depression, anxiety, and hopelessness (all p<0.05). In caregivers of patients with earthquake-related dependency, caregiver burden was associated only with hopelessness (p=0.045).

**Conclusion:**

Informal caregivers of patients with earthquake-related and stroke-related dependency experienced comparable levels of psychological distress, despite differences in caregiving context and disaster-related adversity. The higher frequency of external support among caregivers of patients with earthquake-related dependency may be associated with this pattern, suggesting that family and social support may be relevant to caregiver psychological outcomes in post-disaster settings.

## Introduction

Caregiving is most often provided by family members and involves the support required for patients to maintain daily life. Informal caregivers, who usually do not receive professional training, assist patients with basic needs, provide emotional support, and play an important role in communication with the healthcare system (1, 2). Although institutional care services have increased in Türkiye, caregiving responsibilities are still frequently undertaken by family members (3).

Caregiver burden is a multidimensional concept that encompasses physical strain, emotional exhaustion, social restrictions, and economic difficulties. Numerous studies have shown that this burden increases in conditions such as stroke, Alzheimer’s disease, and other illnesses requiring long-term care (4, 5). In caregivers of stroke patients, elevated levels of depression, anxiety, and hopelessness have been reported, with particular emphasis on the impact of patient dependency level and duration of care on caregiver outcomes (6, 7).

The caregiver stress process model provides a useful framework for interpreting caregiver burden as the result of caregiving demands, contextual stressors, and coping resources. This perspective is particularly relevant after disasters, where caregivers may simultaneously experience traumatic exposure, displacement, financial loss, difficulties in accessing healthcare services, and the sudden responsibility of caring for a newly dependent patient. Within this framework, social support may serve as an important psychosocial resource that influences the relationship between caregiving demands and psychological distress (Saha et al., 2026; Cartaxo et al., 2023; Xu et al., 2021).

During disasters, disruptions in access to healthcare services and the increased needs of dependent individuals may further exacerbate caregiver burden (8). Protecting caregiver mental health is therefore important not only for the continuity of patient care but also for sustaining recovery in the post-disaster period (9). Previous studies following major disasters, including the Great East Japan Earthquake and the Haiti earthquake, have addressed the psychological status of caregivers working in institutional or professional settings (10–12). However, studies examining depression, anxiety, hopelessness, caregiver burden, and post-traumatic stress symptoms among informal caregivers within the same post-disaster context remain limited (13). From a social psychiatry perspective, this gap is important because informal caregiving after a disaster may reflect the combined effects of patient dependency, shared traumatic exposure, family roles, and available social resources.

On 6 February 2023, two earthquakes with magnitudes of 7.8 Mw and 7.5 Mw affected southern and central Türkiye as well as northern and western Syria, causing more than 50,000 deaths (14). Beyond the immediate loss of life and physical destruction, the earthquakes created new caregiving needs for many individuals who became functionally dependent after disaster-related injuries. Some individuals who had been independent before the earthquakes became functionally dependent after the disaster, leading family members to suddenly assume caregiving responsibilities.

This study compared informal caregivers of patients with earthquake-related dependency with caregivers of stroke patients living in the earthquake-affected region. Both caregiver groups were exposed to the psychological effects of the disaster; however, the nature and onset of the care recipients’ dependency differed between groups. This comparison may help clarify how different pathways to newly developed dependency, including neurologically induced disability and sudden disaster-related injury, are associated with caregiver burden and psychiatric symptoms in a shared post-disaster context. To the best of our knowledge, no previous study has compared informal caregivers of patients with disaster-related dependency and those caring for patients with neurologically induced dependency while jointly evaluating caregiver burden, hopelessness, anxiety, depression, and post-traumatic stress symptoms. Therefore, this study aimed to describe and compare caregiver burden and psychiatric symptoms among caregivers of stroke patients and caregivers of patients with earthquake-related dependency following a major disaster.

## Methods

### Study design and participants

This single-center, cross-sectional comparative study included primary unpaid informal caregivers of dependent patients hospitalized in a physical medicine and rehabilitation hospital between June 10, 2023 and August 10, 2023. Data collection started approximately four months after the 6 February 2023 Türkiye–Syria earthquakes, following ethical approval. All care recipients and their caregivers had experienced the earthquakes. During hospitalization, all caregivers stayed with the patients as accompanying family caregivers and were responsible for assisting with their daily care needs. In this study, a primary informal caregiver was defined as the unpaid family member who remained with the patient for at least 18 hours per day during the inpatient rehabilitation period and assumed the main responsibility for assistance with basic activities of daily living. Individuals providing paid caregiving services or only occasional caregiving support were excluded. Participants were eligible if they were older than 18 years of age, had been providing care for 4–6 months, and had sufficient cognitive capacity to understand and complete the questionnaires. Caregivers who were unable to understand and complete the questionnaires, those with schizophrenia spectrum disorders or intellectual disability, and those who self-reported a known psychiatric diagnosis or regular psychotropic medication use before assuming the caregiving role were excluded from the study. Participants were divided into two groups according to the etiology of the care recipient’s dependency: informal caregivers of patients with earthquake-related dependency (Earthquake-CG group, n = 30) and informal caregivers of patients with stroke-related dependency (Stroke-CG group, n = 60).

Earthquake-related dependency was defined as new-onset functional dependency following earthquake-related physical injury in individuals who had been functionally independent before the disaster. The Earthquake-CG group included caregivers of patients with earthquake-related amputation or spinal cord injury.

Stroke-related dependency was defined as new-onset functional dependency due to clinically and neuroimaging-confirmed stroke that occurred after the earthquakes and was not attributable to earthquake-related physical injury. Patients in the Stroke-CG group had been functionally independent at the time of the earthquakes, subsequently developed hemiplegia due to stroke, and required caregiver assistance during the same post-earthquake period. Thus, both groups consisted of caregivers providing care for patients with newly developed dependency within a comparable caregiving duration of 4–6 months.

### Data collection

Sociodemographic data were collected through face-to-face interviews with the caregivers. The Activities of Daily Living Scale was completed by the clinician to assess the functional dependency level of the care recipient. Subsequently, caregivers completed the Beck Depression Inventory, Beck Anxiety Inventory, Beck Hopelessness Scale, Zarit Caregiver Burden Scale, and the PTSD Checklist for DSM-5 (PCL-5). Participants completed the questionnaires individually and were allowed 1–2 days to return the forms. During form collection, unclear items were clarified without directing participants’ responses. Written informed consent was obtained from all participants.

### Sociodemographic and disaster-related data form

In addition to caregivers’ sociodemographic characteristics, the form assessed the degree of kinship to the patient, receipt of external caregiving support, location of care, relocation due to earthquake-related housing damage, difficulties in accessing healthcare services and medications, sleep problems, earthquake-related financial loss, and loss of first- or second-degree relatives during the earthquake.

### Assessment scales

#### Activities of daily living scale

This scale assesses the level of dependency in activities of daily living and covers basic domains such as feeding, dressing, toileting, bathing, continence, and transfer. It is a structured method frequently used to determine patient care needs, and its Turkish validity and reliability have been established (15, 16).

#### Beck depression inventory

The Beck Depression Inventory is a 21-item self-report scale developed to measure the severity of depressive symptoms. Total scores range from 0 to 63. Scores of 0–9 indicate minimal or no depression, 10–16 mild depression, 17–29 moderate depression, and 30–63 severe depression. The Turkish adaptation of the scale has demonstrated high internal consistency and robust psychometric properties and is widely used in clinical populations (17, 18).

#### Beck anxiety inventory

The Beck Anxiety Inventory is a 21-item self-report scale developed to measure the severity of anxiety symptoms. Total scores range from 0 to 63. Scores of 0–7 indicate minimal anxiety, 8–15 mild anxiety, 16–25 moderate anxiety, and 26–63 severe anxiety. The Turkish adaptation has shown that the scale is reliable for clinical use (19, 20).

#### Beck hopelessness scale

The Beck Hopelessness Scale is a 20-item self-report scale that measures negative expectations about the future. Total scores range from 0 to 20. Scores between 0 and 3 are considered within the normal range, scores between 4 and 8 indicate mild hopelessness, scores between 9 and 14 indicate moderate hopelessness, and scores greater than 14 indicate severe hopelessness. The Turkish validity, reliability, and psychometric properties of the scale have been established (21, 22).

#### Zarit caregiver burden scale

The Zarit Caregiver Burden Scale is a 22-item self-report scale that assesses the physical, emotional, social, and economic burden experienced by caregivers of individuals requiring care. Scores range from 0 to 88, with higher scores indicating greater caregiver burden. The Turkish adaptation has been found to be valid and reliable (23, 24).

#### Post-traumatic stress disorder checklist

The PCL-5 is a 20-item self-report scale developed based on DSM-5 diagnostic criteria to assess the severity of post-traumatic stress symptoms. Total scores range from 0 to 80, with higher scores indicating greater symptom severity. The Turkish validity and reliability study of the PCL-5 demonstrated high internal consistency (Cronbach’s alpha > 0.90) and good construct validity in the Turkish sample. A cutoff score of ≥47 has been suggested to indicate clinically relevant post-traumatic stress symptoms; at this cutoff, sensitivity was 0.76 and specificity was 0.69 (25, 26).

### Ethical approval

Ethical approval was obtained from the institutional Clinical Research Ethics Committee on June 8, 2023, with meeting number 128 and decision number 2630. The study was conducted in accordance with the principles of the Declaration of Helsinki. All participants provided written informed consent and were informed of their right to withdraw from the study at any stage.

### Statistical analysis

Data were analyzed using IBM SPSS Statistics for Windows, version 25.0. Categorical variables were presented as numbers and percentages, and continuous variables were presented as mean ± standard deviation. The distribution of continuous variables was assessed using the Shapiro–Wilk test. The chi-square test was used for comparisons of categorical variables. The Mann–Whitney U test was used for between-group comparisons of non-normally distributed continuous variables. Spearman correlation analysis was performed to examine relationships between scale scores. Multivariable linear regression models were used to examine factors associated with caregiver burden and post-traumatic stress symptom scores. A p value of <0.05 was considered statistically significant.

During the study period, all eligible informal caregivers who agreed to participate were consecutively included. The final sample size and group distribution were determined by the number of eligible caregivers available in each diagnostic category during the study period rather than by a predefined sample size target. A sensitivity power analysis was conducted for the achieved sample of 90 participants, with a 1:2 allocation ratio between the Earthquake-CG and Stroke-CG groups. At a two-sided alpha level of 0.05, the achieved sample provided 80% power to detect a between-group difference corresponding to approximately Cohen’s d = 0.63.

## Results

A total of 90 informal caregivers were included in the study: the Earthquake-CG group (n = 30) and the Stroke-CG group (n = 60). Of the caregivers, 66 were female and 24 were male. The mean age of female caregivers was 42.8 ± 15.8 years, whereas the mean age of male caregivers was 50.7 ± 13.2 years, which was significantly higher compared to female caregivers (p = 0.041). Receiving additional support during the caregiving process was less frequent among female caregivers compared to male caregivers (p = 0.011). In the evaluation of caregiver burden, male caregivers had significantly higher scores on the Caregiver Burden Scale than female caregivers (p = 0.026).

In the Earthquake-CG group, receiving external caregiving support was more frequent than in the Stroke-CG group (53.3% vs. 23.3%; OR = 3.76, 95% CI: 1.48–9.56, p = 0.004; effect size = 0.300). However, no statistically significant differences were found between the groups in terms of age, sex, marital status, educational level or duration of caregiving. Detailed data regarding the sociodemographic characteristics of the caregivers are presented in **Table 1**.

**Table 1:** Comparison of sociodemographic characteristics netween the caregiver groups.

Characteristic	Earthquake-CG group (n=30)	Stroke-CG group (n=60)	P	Odds ratio (95%Mean ± SD CI)		Effect size
	n (%)	n (%)				
Sex						
Male	10 (33.3)	14 (23.3)	0.312	1.643 (0.625-4.319)		0.107
Female	20 (66.7)	46 (76.7)				
Place of residence						
Urban	17 (56.7)	33 (55)	0.881	1.070 (0.442-2.588)		0.016
Rural	13 (43.3)	27 (45)				
Marital status						
Single	9 (30)	14 (23.3)	0.563			0.113
Divorced/Widowed	2 (6.7)	8 (13.3)				
Married	19 (63.3)	38 (63.4)				
Education level						
Primary School	12 (40)	40 (66.7)	0.129			0.308
High School	7 (23.3)	9 (15)				
University	11 (36.7)	11 (18.3)				
Receiving External Support	16 (53.3)	14 (23.3)	0.004*	3.755 (1.476-9.556)		0.300
	Mean±SD Med (Min-Max)	Meane ±SD Med (Min-Max)	p	95% CI		Effect size
				Lower	Upper	
Duration of Caregiving (months)	4.97 ± 0.85 5 (4-6)	4.92 ± 0.83 5 (4-6)	0.114	-9.234	3.500	-0.20
Age	46.6±14.0 47.5 (18-70)	49.5±14.5 51 (19-75)	0.353	-0.414	2.081	0.26

Values are presented as n (%) for categorical variables and mean ± standard deviation and median (minimum–maximum) for continuous variables. OR and 95% CI are reported for binary categorical comparisons where applicable. Effect sizes are reported for group comparisons.

CG, caregiver group; n, number; SD, standard deviation; Med, median; Min–Max, minimum–maximum; OR, odds ratio; CI, confidence interval.

*p < 0.05 was considered statistically significant.

Bold values indicate statistically significant p values (p < 0.05).

The distribution of primary caregiving settings during the post-earthquake caregiving period differed significantly between the groups (p = 0.002). In the Earthquake-CG group, care was most commonly provided in the patient’s own home (43.3%), followed by the caregiver’s home (36.7%), container housing (10.0%), and the hospital (10.0%). In the Stroke-CG group, care was provided either in the patient’s own home (71.7%) or in the caregiver’s home (28.3%). The rate of relocation due to earthquake-related housing damage was 66.7% in the Earthquake-CG group, whereas this rate was 5% in the Stroke-CG group (p < 0.001). Caregivers in the Earthquake-CG group reported significantly higher rates of difficulties in accessing healthcare services and medications, sleep problems, and earthquake-related financial losses (p < 0.001). In addition, the rate of losing a first- or second-degree relative during the earthquake was 63.3% in the Earthquake-CG group, compared to 3.3% in the Stroke-CG group (p < 0.001).

No statistically significant differences were found between the groups in terms of patient dependency level (partial and full dependency), as assessed by the Activities of Daily Living evaluation, including functional status in bathing, dressing, toileting, transfer, continence, and feeding domains (p > 0.05). Similarly, no significant differences were observed between the groups regarding caregivers’ levels of depression, anxiety, hopelessness, and post-traumatic stress symptoms, as reflected by the BDI, BAI, BHS, and PCL-5 scores, respectively. The proportion of caregivers with clinically relevant post-traumatic stress symptoms according to the PCL-5 cutoff was also comparable between the Earthquake-CG and Stroke-CG groups (26.7% vs. 21.7%; OR = 0.76, 95% CI: 0.28–2.10, p = 0.597; effect size = 0.056). In both groups, the majority of caregivers reported a mild level of caregiver burden. However, approximately one-third of caregivers in both groups exhibited mild to moderate levels of depressive, anxiety, and hopelessness symptoms. Detailed data regarding these comparisons are presented in **Table 2**.

**Table 2:** Comparison of patient dependency and psychiatric symptom categories between the caregiver groups.

Variable/category	Earthquake-CG group (n=30)	Stroke-CG group (n=60)	p	Odds ratio 95 % CI	Effect size
ADL					
Moderate Dependency Severe Dependency	7 (23.3) 23 (76,7)	14 (23.3) 46 (76.7)	1.000	1.000 (0.355-2.818)	0.000
PCL-5 Score					
Score ≥ 47 Score ≤ 46	8 (26.7) 22 (73.3)	13 (21.7) 47 (78.3)	0.597	0.761 (0.275-2.101)	0.056
ZCBS					
Minimal Mild Moderate Severe	7 (23.3) 23 (76.7) 0 0	11 (18.3) 48 (80.0) 1 (1.7) 0	0.678	–	0.085
BDI					
None Mild Moderate Severe	13 (43.3) 5 (16.7) 7 (23.3) 5 (16.7)	20 (33.3) 22 (36.7) 7 (11.7) 11 (18.3)	0.172	–	0.236
BAI					
Normal Mild Moderate Severe	8 (26.7) 13 (43.3) 5 (16.7) 4 (13.3)	29 (48.3) 14 (23.3) 10 (16.7) 7 (11.7)	0.172	–	0.236
BHS					
Minimal Mild Moderate Severe	9 (30.0) 10 (33.3) 8 (26.7) 3 (10.0)	20 (33.3) 23 (38.3) 11 (18.3) 6 (10.0)	0.834	–	0.098

Values are presented as n (%). OR and 95% CI are reported for binary categorical comparisons where applicable. Effect size values are reported for categorical group comparisons.

ADL, Activities of Daily Living; BAI, Beck Anxiety Inventory; BDI, Beck Depression Inventory; BHS, Beck Hopelessness Scale; CG, caregiver group; CI, confidence interval; n, number; OR, odds ratio; PCL-5, Post-Traumatic Stress Disorder Checklist for DSM-5; ZCBS, Zarit Caregiver Burden Scale.

*p < 0.05 was considered statistically significant.

The relationships between PCL-5 and Caregiver Burden Scale scores and psychiatric symptoms were evaluated for all caregivers. In the Stroke-CG group, PCL-5 scores showed moderate positive correlations with BDI (r = 0.555, 95% CI: 0.354–0.708, p < 0.001), BAI (r = 0.542, 95% CI: 0.338–0.699, p < 0.001), and BHS scores (r = 0.480, 95% CI: 0.258–0.653, p < 0.001). In the same group, ZCBS scores were also positively correlated with BDI (r = 0.443, 95% CI: 0.215–0.625, p < 0.001), BAI (r = 0.309, 95% CI: 0.060–0.522, p = 0.016), and BHS scores (r = 0.477, 95% CI: 0.255–0.651, p < 0.001). In the Earthquake-CG group, ZCBS scores were significantly correlated only with BHS scores (r = 0.382, 95% CI: 0.013–0.657, p = 0.045), while the other correlations did not reach statistical significance. These relationships are presented in **Table 3**.

**Table 3:** Correlations between post-traumatic stress symptoms, caregiver burden, and psychiatric symptom scores in the caregiver groups.

Central variable	Psychological scale	Earthquake-CG r [95% CI) (p)	Stroke-CG r [95% CI) (p)
PCL-5	BDI	0.234 (-0.139-0.548) (0.214)	**0.555 (0.354-0.708) (<0.001)**
	BAI	0.331 (-0.034-0.619) (0.074)	**0.542 (0.338-0.699) (<0.001)**
	BHS	0.247 (-0.138-0.565) (0.205)	**0.480 (0.258-0.653) (<0.001)**
ZCBS	BDI	0.032 (-0.332-0.388) (0.868)	**0.443** (0.215-0.625) **(<0.001)**
	BAI	0.115 (-0.255-0.456) (0.547)	**0.309** (0.060-0.522) **(0.016)**
	BHS	**0.382** (0.013-0.657) **(0.045)**	**0.477** (0.255-0.651) **(<0.001)**

Correlations were analyzed using Spearman’s rho. Values are presented as correlation coefficients with 95% confidence intervals and p values. Statistically significant correlations are shown in bold.

BAI, Beck Anxiety Inventory; BDI, Beck Depression Inventory; BHS, Beck Hopelessness Scale; CG, caregiver group; CI, confidence interval; PCL-5, PTSD Checklist for DSM-5; ZCBS, Zarit Caregiver Burden Scale.

*p < 0.05, **p < 0.01.

An exploratory multivariable linear regression analysis was performed to examine factors associated with caregiver burden. The overall model was statistically significant (F = 2.637, p = 0.007). However, after adjustment for group, age, sex, receiving external support, ADL, PCL-5, BAI, BDI, and BHS scores, none of the included variables showed an independent statistically significant association with caregiver burden (all p > 0.05). Detailed model statistics are presented in **Table 4**.

**Table 4:** Multivariable linear regression analysis of factors associated with zarit caregiver burden scale scores.

Predictor variable	Unstandardized coefficients		Standardized coefficients	p	95% confidence interval for B	
	β	Se	β		Lower	Upper
Constant	36.464	13.052		**0,007**	10.490	62.439
Group	-1.361	3.657	-0.040	0.711	-8.639	5.917
Age	0.178	0.121	0.157	0.146	-0.063	0.419
Sex	-5.294	3.844	-0.145	0.172	-12.943	2.355
Receiving External Support	-4.873	3.808	-0.142	0.204	-12.452	2.705
ADL	-1.678	1.046	-0.164	0.113	-3.760	0.404
PCL-5 score	0.025	0.109	0.027	0.821	-0.192	0.242
BAI	0.158	0.197	0.102	0.423	-0.233	0.550
BDI	0.342	0.191	0.210	0.077	-0.038	0.723
BHS	0.149	0.194	0.082	0.443	-0.236	0.534

The dependent variable was the Zarit Caregiver Burden Scale score. Unstandardized coefficients, standardized coefficients, standard errors, p values, and 95% confidence intervals are presented. Model statistics: R = 0.478, R² = 0.229, adjusted R² = 0.142, F = 2.637, Durbin-Watson = 1.842.

ADL, Activities of Daily Living; BAI, Beck Anxiety Inventory; BDI, Beck Depression Inventory; BHS, Beck Hopelessness Scale; CI, confidence interval; PCL-5, PTSD Checklist for DSM-5; SE, standard error; ZCBS, Zarit Caregiver Burden Scale.

*p < 0.05.

## Discussion

In line with previous studies reporting caregiver burden and psychological distress among caregivers of stroke and trauma patients, this study showed that informal caregivers of dependent patients experienced measurable caregiver burden and clinically relevant psychiatric symptoms after the Türkiye–Syria earthquakes (7, 27). In our sample, caregiver burden was predominantly mild, whereas depressive, anxiety, and hopelessness symptoms were observed in a considerable proportion of caregivers. Despite greater disaster-related adversity in the Earthquake-CG group, caregiver burden and psychiatric symptom categories did not differ significantly between the groups. This finding may indicate that caregiver outcomes in the early post-disaster rehabilitation period should be interpreted not only in relation to the etiology of dependency, but also within the context of care-related demands, shared traumatic exposure, and available caregiving support.

Studies and meta-analyses on informal caregivers show that caregivers are predominantly female and married individuals, and that care recipients are generally spouses, partners, or close relatives (3, 4, 28). The caregiving process is not limited to physical assistance but is defined as a multidimensional role that includes organizing personal care and nutrition, mediating access to healthcare services, transportation, and financial responsibilities. These responsibilities may contribute to the demanding nature of the caregiving role and to the development of caregiver burden (29).

In the literature, female sex, age over 65 years, low educational level, cohabitation with the care recipient, economic difficulties, and comorbid depression have been reported as factors that increase caregiver burden (3, 4, 28–33). However, gender-related patterns of caregiver burden may vary according to caregiving role, cultural expectations, care intensity, and available support. In our sample, male caregivers were older and had higher ZCBS scores, although they reported receiving additional caregiving support more frequently. This finding is noteworthy, as many previous studies have reported higher caregiver burden among women. One possible interpretation is that, in traditional family systems, women may be more familiar with daily caregiving responsibilities through socially expected family roles or previous caregiving experiences within the household. Men, in contrast, may be less accustomed to intensive hands-on caregiving and may experience greater role strain when faced with sudden caregiving demands, particularly in the context of a major disaster. Previous studies based on caregiver stress and role-related frameworks also suggest that caregiver burden is shaped not only by sex itself, but also by role expectations, care demands, social resources, and adaptation to the caregiving role (Swinkels et al., 2018; de Almeida Mello et al., 2017). Therefore, the higher caregiver burden observed among male caregivers in our sample may reflect the interaction between post-disaster caregiving demands and culturally shaped caregiving roles.

In our study, whether the care recipient had experienced a stroke or had become dependent due to the earthquake did not result in a significant difference in caregiver burden. The absence of differences between the groups in terms of sociodemographic variables and duration of caregiving allowed comparisons within a more balanced sample structure and may have reduced the influence of some potential confounding variables.

Newly developed dependency after a major health event may impose substantial emotional and practical demands on informal caregivers, even within the first months of caregiving (4). In this study, moderate to severe levels of depressive symptoms, anxiety, and hopelessness were observed in both caregiver groups at rates ranging from 28.3% to 40%, while most caregivers were classified as having mild caregiver burden.

In our study, caregiver burden among caregivers of stroke patients was predominantly classified as mild. This finding should be interpreted together with the observed depressive, anxiety, and hopelessness symptoms, as caregiver burden and psychological distress may not always show parallel severity levels. Moreover, these studies have reported no significant associations between caregiver burden in stroke caregivers and caregiver age, duration of caregiving, or disease characteristics (6, 30). In our study, the similarity in dependency and functional levels of patients receiving care in both groups may explain why caregivers reported similar levels of depression, anxiety, hopelessness, post-traumatic stress symptoms, and caregiver burden. This may suggest that caregiver burden is more closely related to the scope of care needs and the tasks undertaken than to the diagnostic etiology of dependency itself (34). In this context, the caregiving experience can be considered a dynamic process specific to the relationship between the care recipient and the caregiver.

An increase in caregiver burden has been associated with declines in physical and mental health (28). It has been reported that caregiver burden among caregivers of stroke patients is associated with symptoms of depression and anxiety (35, 36). In our study, however, a moderate positive correlation was found between caregiver burden and hopelessness symptoms among caregivers of patients who became dependent due to the earthquake. The presence of associations between caregiver burden and anxiety, depression, and hopelessness symptoms among caregivers of stroke patients is generally consistent with these findings. However, in the exploratory multivariable linear regression model, none of the included variables showed an independent statistically significant association with caregiver burden after adjustment. This finding suggests that caregiver burden in the early post-disaster rehabilitation period may be related to the combined influence of multiple caregiving-related and psychosocial factors rather than to a single independent determinant.

Similar to the experiences of healthcare providers, the psychological dimension of caregiver burden observed in informal caregivers can be addressed within the framework of secondary traumatic stress. In our study, caregivers of stroke patients, in addition to their caregiving responsibilities, were exposed to one of the largest natural disasters in Türkiye. This combined exposure may partly account for the moderate positive correlations observed between caregivers’ PCL-5 scores and depression (BDI), anxiety (BAI), and hopelessness (BHS) scores (37, 38).

The earthquakes that occurred in Türkiye on February 6 severely affected approximately 16% of the population and 11 provinces, and an estimated 1.5 million people were left homeless. In a study conducted after the Great East Japan Earthquake, evacuated caregivers were reported to experience higher psychological distress compared to non-evacuated caregivers (39). Unlike that study, our study included informal caregivers. Although caregivers were evacuated from their homes, they continued to live with the relatives they cared for and mostly received additional support from their families.

Caregivers of patients who became dependent due to the earthquake had higher rates of evacuation from their pre-earthquake residences compared to caregivers of stroke patients. In addition, this group reported greater difficulties in accessing healthcare services, sleep problems, coping with the loss of relatives, and disaster-related financial hardships. These findings reflect the natural consequences of a large-scale disaster (11).

However, despite these significant differences in adverse experiences, caregiver burden and levels of depression, anxiety, hopelessness, and post-traumatic stress symptoms did not differ significantly between the groups. The higher frequency of external support among caregivers of patients with earthquake-related dependency may be associated with the comparable levels of psychological distress observed between the groups despite greater disaster-related adversity in the Earthquake-CG group. This finding suggests that caregiver mental health may be influenced not only by the etiology of dependency, but also by social resources, family support, and shared caregiving arrangements. Consistent with previous studies showing that caregiving support may reduce perceived burden and that insufficient social support is associated with psychological strain, our results underline the need for sustainable support mechanisms for informal caregivers in post-disaster rehabilitation settings (40–43). In addition, factors such as caregiver sex, degree of disaster exposure, bereavement experiences, socioeconomic hardship, and access to family support may have influenced psychological outcomes in both groups. Therefore, the observed associations should be interpreted within the broader psychosocial context of caregiving after a major disaster.

In a study following caregivers of stroke patients, depressive symptoms after the first year and insufficient caregiver support were identified as major determinants of increased caregiver burden (44). In our study, the support provided to caregivers of patients who became dependent due to the earthquake may decrease over time as daily life gradually returns to its pre-disaster pattern. If support mechanisms weaken while caregiving continues, psychological distress and caregiver burden may increase over time.

Taken together, these findings suggest that support for informal caregivers should not be limited to the acute disaster phase, but should be maintained in a long-term and sustainable manner. Recognition of caregiving labor and stronger institutional support may be important components of post-disaster rehabilitation planning (4, 45).

To reduce the long-term risk of developing mental health problems, it is necessary to ensure continued access to mental health and psychosocial support services for individuals affected by the earthquake beyond the acute phase (14).However, the limited number of studies in the literature focusing on vulnerable groups disproportionately affected by disasters complicates the development of effective solutions and care plans (46, 47).

In a review examining the needs of caregivers of patients with amputation, caregiver needs were grouped under physical health, general health and well-being, supportive care, social support, education and information support, and mental health and psychological support. After a traumatic event such as amputation, caregivers are particularly recommended to be screened for anxiety symptoms within the first month (48).

In the literature, it has been reported that support programs planned according to caregivers’ risk and needs profiles and optionally delivered via telephone have been implemented. In these interventions, short-term psychotherapeutic approaches focus on daily problem-solving and information provision, whereas cognitive behavioral therapy addresses the cognitive and emotional consequences of caregiving (49). Studies examining interventions for caregivers’ mental health have reported that multidisciplinary approaches without psychological content are effective in reducing overall caregiver burden and strengthening perceived social support, but show limited effects on psychological symptoms. In contrast, psychological interventions targeting cognitive behavioral therapy, problem-solving training, and coping skills have been shown to be more effective in improving psychological symptoms (50).

The literature examining the mental states of patients and informal caregivers demonstrates that psychological well-being is mutually interactive. Protecting the mental health of caregivers is critical not only for caregivers themselves but also for the psychological well-being of care recipients and the sustainability of the caregiving process (51). Supporting caregiver well-being in extraordinary situations such as disasters ensures the continuity of this vital support for care recipients and the continuity of healthcare services.

This study has several limitations. First, the relatively small sample size, particularly in the Earthquake-CG group, and the single-center design may limit the generalizability of the findings and reduce the ability to detect small between-group differences. Second, although the main diagnostic categories and caregiving duration were clearly defined, more detailed clinical variables such as stroke severity, lesion characteristics, level of amputation, neurological level and completeness of spinal cord injury, pain severity, and rehabilitation prognosis were not evaluated. Third, although pre-caregiving psychiatric diagnosis and regular psychotropic medication use were assessed through caregiver self-report, these data were not confirmed using structured psychiatric interviews or medical records. Therefore, undocumented or subclinical psychiatric symptoms before caregiving cannot be completely ruled out. Previous trauma exposure, individual coping styles, the severity of personal disaster exposure, and the quality or intensity of social support were also not fully evaluated. In addition, exact weekly caregiving hours were not recorded as a continuous variable; therefore, caregiving intensity could not be analyzed quantitatively beyond the operational definition of primary caregiving. Fourth, the cross-sectional design does not allow causal inferences regarding the relationships between caregiving context, external support, caregiver burden, and psychiatric symptoms. Because data were collected within a single 4–6-month post-disaster caregiving window, this study could not evaluate temporal changes in caregiver burden or the persistence, delayed worsening, or recovery of post-traumatic stress symptoms over time. Nevertheless, the inclusion of two caregiver groups with newly developed patient dependency and comparable caregiving duration provides clinically relevant information on informal caregiving in a post-disaster rehabilitation context.

## Conclusion

The 6 February 2023 Türkiye–Syria earthquakes highlighted that informal caregivers may face not only caregiving demands but also multiple psychosocial stressors in the post-disaster period. In this study, caregiver burden and psychiatric symptoms were comparable between caregivers of patients with earthquake-related dependency and those caring for patients with stroke-related dependency, despite greater disaster-related adversity in the Earthquake-CG group. The higher frequency of external support among caregivers of patients with earthquake-related dependency may be associated with this pattern and suggests that family and social support may be relevant to caregiver psychological outcomes.

These findings underline the need to consider informal caregivers as a distinct psychosocial risk group in disaster preparedness, rehabilitation planning, and post-disaster mental health services. Support for caregivers should not be limited to the acute disaster phase but should be structured, sustainable, and integrated into long-term rehabilitation and psychosocial care. Future longitudinal studies with larger samples are needed to clarify the long-term trajectories of caregiver burden, psychiatric symptoms, and the potential role of social support after disasters.

## Data Availability

The raw data supporting the conclusions of this article will be made available by the authors, without undue reservation.
